# Reliability of Family Proxy Data for Studies of Malignant Mesothelioma: Results from the ATSDR Pilot Surveillance

**DOI:** 10.1155/2013/325409

**Published:** 2013-03-28

**Authors:** Natalia Melnikova, Jennifer Wu, Wendy Kaye, Maureen Orr

**Affiliations:** Agency for Toxic Substances and Disease Registry, Division of Toxicology and Health Sciences, Buford Highway, Atlanta, GA 30341, USA

## Abstract

*Objective*. To evaluate the validity of proxy interviews in obtaining information on persons with rapidly fatal diseases such as malignant mesothelioma (MM). *Methods*. Persons with MM diagnosed in 2002 through 2005 in New York and New Jersey and 1997–2004 in Wisconsin were eligible for inclusion in the project. Persons with MM and their family member proxy were interviewed using the same questionnaire designed by ATSDR to collect information on potential direct or indirect occupational and environmental exposure to asbestos, genetic, and health related malignancy predisposition, and exposure to tobacco products. Descriptive statistics and the McNemar/Durkalski test were used to analyze 33 matched pairs. *Results*. The overall study confirmed a generally high ability of proxies to give interviews of comparable quality and completeness when asked dichotomous questions. The reliability of information collected from proxies varied by topic and family relationship. *Conclusions*. Family proxy interviews, using dichotomous responses, can serve as an acceptable source of information about health and exposure-related risk factors for MM.

## 1. Introduction

Mesothelioma is an uncommon, malignant neoplasm that arises from mesothelial tissue usually in the pleura, less often in the peritoneum, and rarely elsewhere. Prognosis for malignant mesothelioma (MM) is poor, and the tumor has a median latency period of 32 years from the time of exposure to causative agents [1, 2]. Exposure to asbestos is considered the most important cause of MM [3, 4]. In diseases with potential occupationally or environmentally related causes, such as mesothelioma, exposure identification is critical for the development of preventive public health strategies.

Once diagnosed, the median length of survival for persons with MM varies from 5.9 to 11 months [5–7]. The ability to interview persons with newly diagnosed MM is limited because of the very short survival period. Proxy interviewing is a commonly used method of obtaining information on persons with rapidly fatal diseases [8, 9]. However, some studies have shown that data received from proxies may be inaccurate or require the review of additional information sources [10–12].

For this investigation, we collected data on the persons potential direct or indirect occupational and environmental exposure to asbestos, genetic, and health related malignancy predisposition, and exposure to tobacco products which does not cause mesothelioma by itself but may complicate the person's chances of contracting a disease [13]. We collected this information from both the person with MM and a family member designated as his or her proxy. This analysis evaluates the validity of proxy interviews.

## 2. Methods

MM was defined as an International Classification of Disease (ICD) Oncology (ICD-9 and ICD-10) histology codes 9050 through 9053. Persons with MM diagnosed in 2002 through 2005 in New York and New Jersey and 1997–2004 in Wisconsin were eligible for inclusion in the project. Persons with MM were ascertained through State Cancer Registries. Additionally, in New York and New Jersey, names, phone numbers, and addresses of persons with newly diagnosed mesothelioma were collected through monthly visits and phone calls to hospitals where the majority of mesothelioma patients were observed and treated. As soon as persons with MM were identified and were determined to be alive, we contacted their physician to obtain the written or verbal consent to contact his/her patients. If consent was received, we sent an introductory letter inviting them to participate in this investigation. Then we contacted them by phone to obtain verbal consent and interview. During the interview, we asked them to identity a family member they knew them best and who could give the most detailed information in a proxy interview. At a later point in time, which varied, the proxies gave verbal consent and were interviewed. 

Trained interviewers conducted telephone interviews using a questionnaire designed by ATSDR. The interview focused on the respondents' and their family's exposure history and medical history, and respondents' smoking history. The Environmental Protection Agency (EPA) list of jobs/activities potentially associated with exposure to asbestos was adapted for this project to assess the exposure history [14].

There were a total of 77 questions on five topics; 73 of these questions for which paired responses were a dichotomous “Yes” or “No,” were analyzed. Four nondichotomous questions, were excluded from the analysis. Pairs where one or both respondents had missing, “Don't know” or “Refused” answers, were also excluded from the dichotomous analysis. Analysis included 15 case exposure history questions, 24 case medical history questions, 14 family exposure history questions, 13 family medical history questions, and 7 smoking history questions. The relationship of the proxy to the case was categorized as spouse, child, sibling, or other (mostly parents). To evaluate the response differences, we stratified the response data by the relationship subgroups during the analysis.

## 3. Statistical Analysis

In 1947, McNemar introduced a test for matched-pair data with a dichotomous response. Since then, this test has become a commonly used method for analyzing paired binary response data as correlated proportions [15]. In our analysis, these procedures refer to the case and the proxy, respectively. The McNemar test assumes independence among the paired responses. Our study evaluated matched-pair responses to five topics. Each topic included a varied number of questions. Each case/proxy pair contributed more than one paired binary response to each topic. Therefore, the independence assumption no longer existed, and application of the McNemar test could result in inflating paired responses and underestimating standard error.

Durkalski et al. [16] proposed an adjusted McNemar test for analysis of clustered matched-pair data. This adjusted McNemar test allows testing the agreement of multiple paired responses between the respondents and their proxy with no inflated information. The adjusted chi-square value, [*χ*_*v*_^2^] was calculated as

(1) $$\begin{array}{c} \chi_{v}^{2}=\frac{{\left(\sum_{k=1}^{K}\left(b_{k}-c_{k}\right)/n_{k}\right)}^{2}}{\sum_{k=1}^{K}{\left[\left(b_{k}-c_{k}\right)/n_{k}\right]}^{2}}, \end{array}$$

where *b*_*k*_ = ∑_*j*=1_^*n*_*k*_^ *b*_*jk*_ and *c*_*k*_ = ∑_*j*=1_^*n*_*k*_^ *c*_*jk*_, and *b*_*jk*_, *c*_*jk*_ = 0 or 1 for all *j* questions in the *k*th matched-pair responding. The *n*_*k*_ is the valid count of paired binary response. The *K* is the total number of matched pairs in the study. The *b*_*k*_ and *c*_*k*_ are the summation of the two types of discordant of *b*_*jk*_ and *c*_*jk*_, respectively. We used the McNemar/Durkalski test to adjust the correlated multiple paired responses on each topic. The null hypothesis of this test is that the two marginal probabilities are the same; that is, there is no difference between the responses from matched pairs. If the *P* value was less than 0.05, we rejected the null hypothesis. The data were analyzed using SAS version 9.3.1 analytical software (Cary, NC).

## 4. Results

Total of 33 paired interviews (eight paired interviews in New Jersey, ten paired interviews in New York, and 15 paired interviews in Wisconsin) were collected. Demographic characteristics of respondents are presented in Table 1. Respondents were mostly white males, not of Hispanic or Latino origin, married, and over 60 years old with a high school or college pregraduate education.

The familial relationship of the proxy is presented in Table 2. There were more spouses than any of the other categories combined (51.4%). Of the proxies, 85% knew the case over 30 years.

Responses of the 33 matched pairs are presented in Table 3. Spouses had the best data completion, with only 3 questions being unanswered. Respondent medical and family medical questions were the most difficult to answer with 3.8% and 4.2% unknown/missing, respectively. Spouses performed the best in exposure questions (89.8% agreement) and respondent and proxy medical history questions (94.1% and 90.0% agreement accordingly). Parents performed the best at family exposure questions (95.7% agreement). Children showed better agreement on smoking history (90.5%). However, the McNemar/Durkalski test discovered no statistically significant heterogeneity between paired responses regardless of relationship and the question's topic. The only exception was a chi-square value 5.57 (*P* = 0.02) for smoking history, showing statistically significant disagreement between paired responses. This was statistically significant only for spouses (chi-square value 5.91, *P* = 0.02), although they answered the same percent of questions correctly as siblings and parents.

## 5. Discussion

Of the 33 paired interviews obtained from New Jersey, New York, and Wisconsin, we generally found agreement across the topics regardless of the familial relationship. The exception was smoking history, an area in which paired responses statistically differed. However, the larger number of spouses in the cohort contributed to the statistical significance. The real percent disagreement was not really that much more for spouses than for other subgroups of proxies. The generally high ability of surrogate responders to give interviews of comparable quality and completeness of exposure data was reported by Nam et al. [17], Pickle et al. [18], and Cordiero [11]. Similar results were found by Campbell et al. [19], who applied the polytomous logistic regression models analyzing the utility of proxy versus index respondent information in a population-based case-control study of rapidly fatal cancers. Polytomous logistic regression models found only a few examples of meaningful heterogeneity among all variables including the cigarette smoking model.

The biggest limitation of this study was the small number (33) of paired interviews available for the analysis. For example, we may speculate that siblings and others that have borderline *P* values for each question group might show significant difference with a larger number of pairs. The interpretation of results with regard to the accuracy of proxy interviews could be affected by the biased nature of the sample. For example, respondents were asked what family member would provide the best information. Additionally respondents may have had a somewhat longer survival from time of diagnosis than those not able to participate, perhaps because they were younger or had a stronger social support network of relatives. In addition, other factors, such as pending litigation and communication between pairs about the interview, may affect the accuracy. This theory could support relatively high agreement for these dichotomous questions. The more detailed multilevel responses were not analyzed because they did not show good agreement or were difficult to meaningfully compare answers.

## 6. Conclusions

Obtaining exposure information on persons with rapidly fatal diseases such as MM and other aggressive malignancies requires using alternative methods of collecting information about risk factors. Our study indicates that family proxy interviews, using dichotomous responses, can serve as an acceptable source of information about health and exposure-related risk factors for MM. 

The reliability of information received from proxies was generally high but varied by topic and familial relationship, all of which need to be taken into consideration, particularly in diseases strongly associated with smoking.

Although the number of pairs available for analysis was small, these data suggests proxy interviews adequately answered big-picture questions regarding exposure. Future studies should explore whether more accurate information can be obtained through the use of interviews with multiple proxies per case.

## Figures and Tables

**Table 1:** Demographic characteristics of respondents with malignant mesothelioma.

Demographic variables	*N *	%
Sex		
Male	23	69.7
Female	10	30.3
Race		
White	31	93.9
Black	0	0
Other	2	6.1
Ethnicity		
Hispanic or Latino/a	2	6.1
Not Hispanic or Latino/a	31	93.9
Marital status		
Married	27	81.8
Widowed	3	9.1
Separated/divorced	1	3.0
Never married	2	6.1
Age at time of interview		
<60 years	12	36.4
60–79 years	17	51.5
80+ years	4	12.1
Education		
<High school	5	15.2
High school some college	25	75.7
Graduate/postgraduate	2	6.1
Other	1	3.0

**Table 2:** Proxy relationship to respondent and time acquainted.

	*N *	%
Relationship to the case		
Spouse	17	51.4
Child	6	18.2
Sibling	5	15.2
Other/parent	5	15.2
Length of relationship		
30 years and less	5	15.2
>30 years	28	84.8

**Table 3:** McNemar/Durkalski statistics for paired responses, by type of relationship and type of question.

Question set	Proxy type	Paired responses						McNemar/Durkalski Test	
		Agreed		Disagreed		Missing/unknown*			
		#	%	#	%	#	%	Chi-square	*P* value
	Spouse	229	89.8	24	9.4	2	0.8	1.48	0.22
Respondent exposure	Child	76	84.4	14	15.6	0	0.0	1.87	0.17
(15 questions)	Sibling	64	85.3	9	12.0	2	2.7	0	1
	Other/parent	62	82.7	13	17.3	0	0.0	0.14	0.71
	Total	**431**	**87.1**	**60**	**12.1**	**4**	**0.8**	**0.15**	**0.70**

	Spouse	384	94.1	24	5.9	0	0.0	1.14	0.29
Respondent medical	Child	119	82.6	7	4.9	18	12.5	2	0.16
(24 questions)	Sibling	98	81.7	10	8.3	12	10.0	1.92	0.17
	Other/parent	103	85.8	17	14.2	0	0.0	3	0.08
	Total	**704**	**88.9**	**58**	**7.3**	**30**	**3.8**	**2.53**	**0.11**

	Spouse	213	89.5	25	10.5	0	0.0	1.37	0.24
Family exposure	Child	71	84.5	13	15.5	0	0.0	0.02	0.88
(14 questions)	Sibling	58	82.9	12	17.1	0	0.0	3	0.08
	Other/parent	67	95.7	3	4.3	0	0.0	1	0.32
	Total	**409**	**88.5**	**53**	**11.5**	**0**	**0.00**	**1.30**	**0.25**

	Spouse	199	90.0	22	10.0	0	0.0	0.02	0.89
Family medical	Child	64	82.1	8	10.3	6	7.7	1.29	0.24
(13 questions)	Sibling	47	72.3	6	9.2	12	18.5	0.67	0.41
	Other/parent	56	86.2	9	13.8	0	0.0	3	0.08
	Total	**366**	**85.3**	**45**	**10.5**	**18**	**4.2**	**0.95**	**0.33**

	Spouse	102	85.7	16	13.4	1	0.8	5.91	0.02
Smoking	Child	38	90.5	4	9.5	0	0.0	1.8	0.18
(7 questions)	Sibling	30	85.7	5	14.3	0	0.0	0.67	0.41
	Other/parent	30	85.7	5	14.3	0	0.0	3.57	0.06
	Total	**200**	**86.6**	**30**	**13.0**	**1**	**0.4**	**5.57**	**0.02**

*Missing or unknown answers were excluded from the McNemar/Durkalski statistic calculation.
